# Involvement of global coordinative structure in achieving the local pendulum swinging task

**DOI:** 10.1371/journal.pone.0262525

**Published:** 2022-02-22

**Authors:** Yusuke Yagai, Akito Miura, Hiroyuki Mishima, Nobuhiro Furuyama

**Affiliations:** 1 Waseda University Advanced Research Center for Human Sciences, Tokorozawa City, Saitama, Japan; 2 Waseda University, Faculty of Human Sciences, Tokorozawa City, Saitama, Japan; University of Minnesota, UNITED STATES

## Abstract

In the literature on inter-limb coordination, the coordination among ‘‘focal’’ body parts (i.e., the two limbs) directly engaged in a pendulum swinging task has been studied by immobilizing other body parts to reduce “noise,” while putting aside questions of how one maintains posture while performing the task. However, in practical performance of musical instruments, for example, performers must coordinate different body parts in sync with the music while maintaining the whole body’s balance. This study demonstrates the effectiveness and necessity of understanding inter-limb coordination in whole-body coordination. Participants were asked to move two pendulums either in sync or alternatively with metronome beeps under two conditions: immobile (fixed forearms) and mobile (forearms not fixed). The explorative analyses focused on whether and how coordinative structures emerged and whether the degree of task achievement differed according to the phase mode, frequency, and mobility conditions. The motion similarity and phase difference between different parts and the pendulums showed that task-specific coordinative structures emerged in both immobile and mobile conditions. In the in-phase mobile condition, the emergent coordinative structure may have improved task achievement, shown by the phase difference between the left and right pendulums. These findings suggest that the global coordinative structure is involved in achieving the local pendulum swinging task.

## Introduction

In the performance of a musical instrument, dance, etc., performers are required to coordinate different body parts in sync with the rhythm of the music while simultaneously controlling posture to maintain the balance of the whole body. For example, in drum playing, wherein one beats the instruments by using sticks in both hands and kicking the foot pedals, a skilled drummer employs the whole body, including the head and trunk. Such control in musical performance has been studied while concentrating on “focal” inter-limb coordination in the framework of the dynamical systems approach (DSA).

DSA considers the body as an open non-equilibrium system and provides a mathematical model to describe body movements as a self-organizing phenomenon affected by intrinsic and extrinsic constraints. Studies by Kelso et al. showed that control parameters such as frequency affect the behavior of periodic inter-limb coordination synced with metronome beeps [1]. For example, a less stable coordination pattern (e.g., maintaining the relative phase of the left and right limbs at 180 degrees, i.e., anti-phase) spontaneously undergoes a phase transition to a more stable coordination pattern (e.g., 0 degree, i.e., in-phase) when a certain critical point in frequency is exceeded. Thus, DSA treats local body parts as oscillators and identifies variables that determine the behavior of the oscillators as a group. Therefore, in these studies, to avoid introducing unwanted noise into the data to treat the motion of focal body parts as the behavior of two oscillators, the experimental settings are prepared such that no mechanical interactions between the limbs and other body parts are permitted. Specifically, the forearms are immobilized and fixed to the tabletop to prevent them from interacting with other body parts, such as the torso, and the task is performed only with the wrists that are allowed to move freely [2].

To explain and predict human periodic motion in dynamic terms, Haken, Kelso, and Bunz [3] proposed a mathematical model called the Haken-Kelso-Bunz (HKB) model to express an order underlying the inter-limb coordination (represented by the relative phase) in terms of a potential function and differential equations with task frequency as a control parameter. This model shows that when the motion of the body can be regarded as a simple harmonic motion, the behavior of the two oscillators exhibits multi-stable points in the phase space in the in-phase/anti-phase state in the system, with frequency as a control parameter. Since bimanual coordination has been studied from the HKB perspective, the same approach has been adopted in the design wherein the relevant body parts are fixed in experimental demonstrations of the model to make the oscillations closer to a simple harmonic motion. The model has been extended to a wide range of phenomena beyond the symmetrical inter-limb coordination by adding new terms (e.g., the ΔΩ term [4] and stochastic noise term [5]) that include coordination between asymmetric body parts (e.g., arm-leg [6] and ankle-hip [7]) and inter-personal coordination (e.g., two-person [8, 9] and eight-person coordination [10]).

Although previous studies have successfully elucidated the inter-limb coordination in terms of the HKB model by limiting their observation to fully controlled experiments, they have not addressed more realistic situations (e.g., playing a musical instrument and dancing) in which performers are required to achieve tasks such as postural control, as mentioned at the beginning of this paper. It is thus essential to understand the local coordination movements between body limbs (e.g., relative phase of the left and right limbs) as not independent from the other body parts but related to postural control of the whole body, including elbows, shoulders, trunk, and head.

Based on the idea of Bernstein [11], motor control can be considered as consisting of a goal-directed “focal” movement and “postural” control [12, 13]. For example, postural perturbation associated with raising the upper limb in a sitting posture when pushing a lamp on a tabletop is compensated for by counterbalancing the opposite body part, which allows the task to be performed efficiently [14].

To fully understand more realistic movements such as playing a musical instrument or dancing, it is necessary to examine whether and how the global coordinative structure in relation to the focal body movements emerges to maintain the posture while allowing one to achieve the task when the body is unconstrained. The present study aims to explore and describe this.

Thus, this exploratory study aims to identify global coordinative structures that emerge in the pendulum-swinging task using the left and right limbs (which is usually considered a merely local coordination task) and to specify the relationship between the global structure and local task achievement. To achieve this goal, while keeping factors such as relative phase modes (in-phase and anti-phase) and task frequencies (six levels) more or less the same as in previous studies, we conduct an experiment to see the effects of the “mobility” factor, which consists of the following two conditions: (1) the “immobile” condition, wherein the participants’ forearms are fixed to the tabletop and their mechanical interaction with the other body parts are not allowed, (2) and the “mobile” condition, wherein the forearms are unconstrained and their mechanical interaction with the other body parts is allowed. All tasks are performed while the participants are seated on a stool.

The data are analyzed from two perspectives: whether and how the global coordinative structure emerged and the degree of task achievement. Regarding the global coordinative structure, the motion similarity (i.e., first peak value of cross-correlation) and phase difference (i.e., mean of the relative phase) of the pendulum to each body part (i.e., the vertex, C7, both acromia, elbow joints, and wrist joints) are calculated and compared among conditions. Regarding the degree of task achievement, the mean and standard deviation (SD) of the phase difference between the left and right pendulums are calculated and compared among the conditions. We analyze the motion data of the anterior-posterior axis, where the periodicity of the pendulum is more clearly shown than that of the other axes.

We consider that the mobile condition enables more flexibility for the human-pendula system to reorganize different demands in different conditions in the task frequency and relative phase mode. Accordingly, in the mobile condition, compared with the immobile condition, we predict that movement is more organized in a way such that the moving hands and/or pendulums are not only compensated for by but are also embedded in the global coordinative structure, including forearms, upper arms, trunk, and head. Additionally, we predict that the global coordinative structure varies depending on the task frequency and relative phase mode, and the degree of task achievement is higher in mobile conditions than in immobile conditions.

## Materials and methods

### Participants

Eighteen students from Waseda University participated in this experiment. They were all male and right-handed students, without any injuries or other health problems to participate this experiment (mean age = 23.3 years, *SD* = 1.27 years; mean height = 171.43 cm, *SD* = 7.36 cm). They were all recruited from the School of Human Sciences or Graduate School of Human Sciences, Waseda University.

### Apparatus

#### Pendulum

The pendulums used in this experiment were each made with a wooden rod (length = 45 cm, weight = 108 g, radius = 2 cm), metal weight (191 g), and bicycle handlebar grip (length = 9 cm, weight = 37 g, radius = 3 cm). When the pendulums were held by a participant, a medical supporter was wound tightly around the holding hand to avoid creating a space between the pendulum and the palm.

#### Armrest

A desk with a tabletop, whose height was adjustable between 72 and 123 cm was used as an armrest (H2B, Flexispot).

#### Stool

A stool, whose height could be adjusted, was used to allow the participant to be seated (D-1000N, Pearl Inc.).

#### Metronome

A metronome was used to maintain the tempo of the experimental task (DB-60, Boss).

#### Headphone

A pair of headphones was used for the participant to listen to the metronome sound (MDR-CD900ST, Sony Inc.).

#### Motion capture system

A three-dimensional opto-electronic motion-capture system (OptiTrack, Natural Point Inc.) with 12 cameras (camera version: Flex 3) was used to record at a sampling rate of 100 Hz the movements of the pendulum and the participant’s body parts (see the section on procedure for the details) where the reflective markers were attached.

#### Data acquisition system

A data acquisition system (TRIAS, DKH, Inc.) was used to synchronize and record motion and metronome data that underwent analog to digital conversion (100 Hz).

### Design and task

The participants performed a pendulum-swinging task using both hands. They were instructed to move a pair of pendulums each held by their left and right hands, to the beat of the metronome beeps. The experimental design had a 2 × 6 × 2 within-participant design; the first factor was the mobility of the forearms (mobile vs. immobile); the second factor was the frequency of swinging movements (6 levels, from 1.5 Hz to 2.5 Hz in steps of 0.2 Hz); and the third factor was the relative phase (in-phase and anti-phase). In each trial, the measurement was started after the swinging movements reached 10 cycles, and the measurement lasted till we obtained 30 movement cycles for each trial (e.g., 12 s in the 2.5 Hz condition and 20 s in the 1.5 Hz condition).

To avoid unnecessary variances in the data, and to counterbalance the effect of order by fastening or loosening the Velcro tapes in every trial (detailed below), nine participants performed the mobile condition first and the immobile condition later, while the remaining nine participants performed the tasks in the reverse order. The order of the other 12 conditions was randomized for each block.

### Procedure

**Fig 1:**
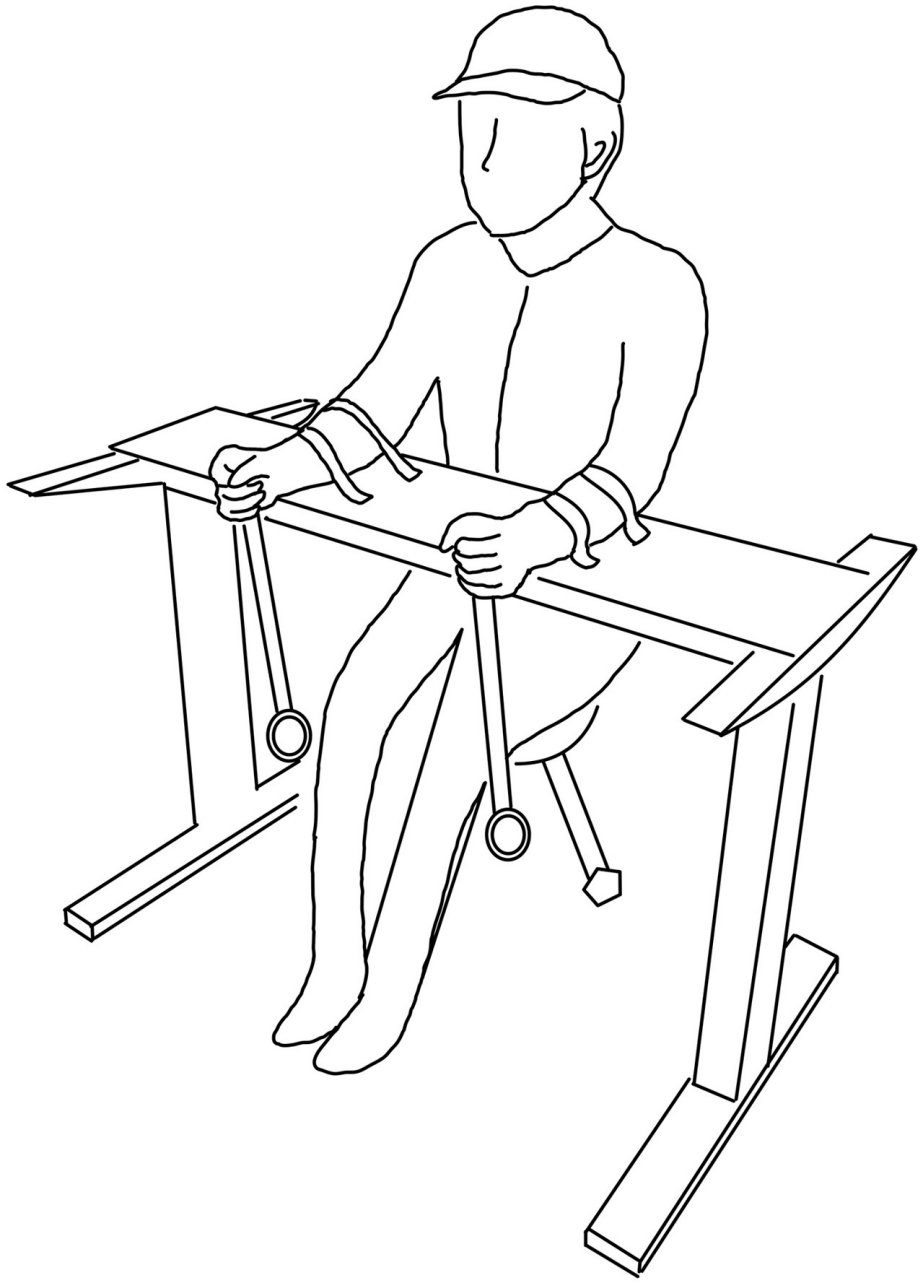
Experimental setup for the immobile condition.

On arrival, the participants were informed of the procedures of the experiment and signed an informed consent form on their own will. The participants then changed into a motion capture suit and had a cap on (OptiTrack, Natural Point Inc.) with reflective markers on the vertex, C7, both acromia, elbow joints (olecrana of both ulnae), and wrist joints (heads of both ulnae). Meanwhile, the height of the eyes in the upright seated position was marked on the wall, and the participants were instructed to keep their eyes on the marker as they performed the task. The height of the tabletop was adjusted such that the forearms were horizontal. During each trial, the participants were instructed to synchronize the movements of the pendulum to the presented metronome beeps as accurately as possible. In the in-phase condition, the tips of the pendulums came to the point closest to the body as the metronome beep sounded (i.e., abduction-of-both-hands-on-the-beat). In the anti-phase condition, the tip of the right-hand-held pendulum came to the point closest to the body, and the tip of the left-hand-held pendulum came to the point farthest from the body as the metronome beep sounded (i.e., abduction-of-right-hand-on-the-beat and adduction-of-left-hand-on-the-beat). The participants were further instructed to move the pendulum as straight, continuously, and smoothly as possible under all conditions. The participants practiced until they could perform the task under all conditions, as instructed. In the immobile condition, the forearms were fastened with two Velcro tapes tightly to the armrests and were adjusted to be horizontal (Fig 1). In the mobile condition, the armrest (and the desk as a whole) was removed from the participants, but they were instructed to keep the forearms horizontal during the task. In consideration of the participants’ fatigue, a break of a few minutes was taken between the trials.

This study was reviewed and approved by the Ethics Review Committee on Human Research of Waseda University (2017–140) and was performed in accordance with the Declaration of Helsinki.

### Data analyses

#### Pre-processing

In the data analysis, we used the time series of the anterior-posterior motion of the body and the pendulums. The first 10 cycles were discarded to remove the transient fluctuations in the data. The remaining data were bandpass-filtered using a 0.1–10 Hz Butterworth filter (2nd order, bi-directional) and z-scored.

#### Relationship between the pendulum and body parts

Because we wanted to know whether and the extent to which the left and right pendulums and body parts are related to each other, the first peak value of cross-correlations (as a measure of similarity) and relative phases (as a measure of phase difference) of all of these in relation to the right and left pendulums were calculated. The lag in calculation of the cross-correlations was set to one cycle before and after. The relative phases were obtained as follows. The motion data underwent the Hilbert transformation to obtain the phase angles. Then, the continuous relative phases of each body part relative to the left and right pendulums were calculated. Because we only focused on the phase difference in this analysis, we calculated the average of the relative phases. Therefore, 18 relative phases and 18 cross-correlation coefficients were obtained (right pendulum: nine pairs; left pendulum: nine pairs; total 18 pairs).

#### Visualization of the global coordinative structure

**Fig 2:**
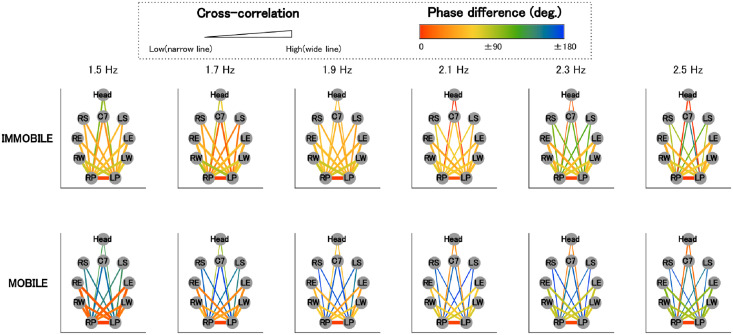
Visualization of the global coordinative structure (in-phase) (*N*=18). This shows the global coordinative structure (*N*=18). Mean first peak values of the cross-correlation and phase difference between the time series of anterior-posterior motion of the pendulum and each body are shown as lines projected onto the topologically arranged body parts (gray circles). The degree of cross-correlation is expressed by the thickness of the line and the phase difference by color gradients. RP represents the R-pendulum, LP L-pendulum, RW R-wrist, LW L-wrist, RE R-elbow, LE L-elbow, RS R-shoulder, and LS L-shoulder.

**Fig 3:**
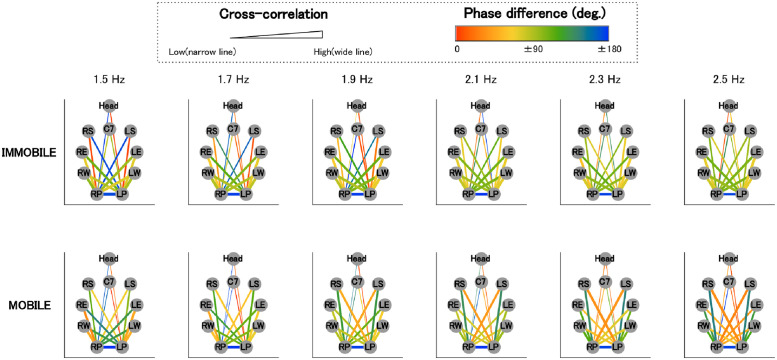
Visualization of the global coordinative structure (anti-phase) (*N*=18). Fig 3 represent the same information as Fig 2, except that the data are for anti-phase.

To visualize the common coordination among participants, the above two indices were averaged and presented as lines projected onto the topologically arranged body parts (see Figs 2 and 3). The degree of cross-correlation is expressed by the thickness of the line, and the phase difference by color gradients.

#### Degree of task achievement

The degree of task achievement and phase stability of the left-right pendulums were evaluated based on the phase shift and SD*ϕ*, respectively. Continuous relative phases (*ϕ*) of the left and right pendulums were computed using the same method as in the previous section (*ϕ* = *θ*_*right*_ − *θ*_*left*_). The phase shift and the absolute value of the phase shift were calculated using circular statistics [15, 16]; the phase shift is the difference between the mean value of the relative phases (mean*ϕ*) and the prescribed relative phase mode (in-phase = 0 degree, anti-phase = 180 degrees). Additionally, the absolute value of the phase shift was obtained. The phase shift and its absolute value were meant to indicate the extent to which participants achieved the task. Meanwhile, SD*ϕ*, calculated using circular statistics [15, 16], represents the stability of the phase difference between the left and right pendulums.

#### Statistics

To examine the main effect and interaction of the two factors on each index (phase shift, absolute value of phase shift, SD*ϕ*, and relative phase between body parts), the Harrison-Kanji test was employed, and the two-way ANOVA was applied to the Fisher z-transformed cross-correlation coefficients. The Harrison-Kanji test is used for the ANOVA of angular data, allowing the examination of two-way main effects and interaction [17]. The phase mode was not included as a factor in the statistical tests because the motions involved in the two-phase modes are qualitatively different; thus, a two-factor test was performed for each phase mode. When the interaction was significant, simple main effect tests were conducted. These included the Watson-Williams test for angular data and a one-way ANOVA for correlated data. If only the main effect (mobility or frequency) was significant, a post-hoc t-test (for angular data, Watson-Williams test) was conducted to examine the difference between conditions. The p-values were corrected using the Holm method (p = 0.05).

## Results

### Visualization of the global coordinative structure

In Figs 2 and 3, the mean first peak values of cross-correlation and phase difference between the time series of the anterior-posterior motion of the pendulum and each body are shown as lines projected onto the topologically arranged body parts (gray circles).

#### In-phase

We see that cross-correlations in the in-phase condition (Fig 2) were higher (lines are thicker) in the immobile condition than in the mobile condition.

Regarding phase differences in the in-phase condition (Fig 2), we see that while the phase differences between the pendulum and body parts close to the pendulum (i.e., wrist and elbow) changed from approximately 90 to 60 degrees (light green to yellowish light green) as frequency increased, in the mobile condition, they changed from approximately 0 to 90 degrees (red to light green). Moreover, phase differences in the in-phase condition (Fig 2) between the pendulum and remote body parts (i.e., shoulder and C7), except for the head, changed from 45 to 90 degrees (orange to light green) in the immobile condition, while they remained approximately between 170 and 180 degrees (slightly greenish blue to blue) in the mobile condition, regardless of frequency. Regarding phase differences between the pendulum and the head, they changed from approximately 130 to 20 degrees (green to orange) in the mobile condition, while they changed from approximately 90 to 0 degrees (light green to red) in the immobile condition.

#### Anti-phase

For the anti-phase, as Fig 3 shows, cross-correlations between the shoulder and the pendulum are consistently low (thin lines), regardless of the frequency in the immobile condition. However, they became higher (thicker lines) as frequency increased in the mobile condition. Additionally, cross-correlations were lower (thinner lines) for C7 and the head than for the shoulders, regardless of mobility.

Fig 3 also shows that phase differences for body parts close to the pendulum in the immobile condition were around 90 degrees (light green) for the wrists and around 80 degrees for the elbows, regardless of frequency. In contrast, in the mobile condition, phase differences between the body parts and the pendulum of the same side (e.g., R-pendulum and R-wrist) changed from approximately 45–130 degrees (orange to light green), and those of the opposite side (e.g., R-pendulum and L-wrist) changed from approximately 130–45 degrees (light green to orange) as frequency increased.

As for body parts remote from the pendulum (shoulder, C7, and head), while there were unignorably large individual differences for phase differences between C7/head and the pendulum, a characteristic pattern of phase differences between the shoulder and the pendulum was observed in both the mobile and immobile conditions (Figs 5 and 7).

In the immobile condition, at low frequencies (1.5 Hz and 1.7 Hz), phase differences between the shoulder and the pendulum of the same side were close to 0 degree (red), and those of the opposite side were close to 180 degrees (blue). However, as the frequency increased, phase differences between the shoulder and the pendulum, regardless of sides, became approximately 90 degrees (light green), similar to the patterns observed for the wrists and elbows.

In contrast, in the mobile condition, phase differences between the shoulder and the pendulum of the same side (e.g., R-pendulum and R-shoulder) were closer to 180 degrees (blue), and those of the opposite side (e.g., R-pendulum and L-shoulder) come close to 0 degree (red) as the frequency increased.

### Motion similarity between the pendulum and body parts

#### In-phase

**Fig 4:**
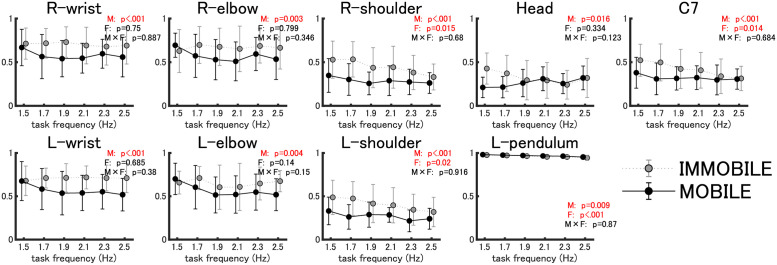
Motion similarity between the pendulum and body parts (in-phase) (*N*=18). This shows the mean and standard deviation (error bars) of the first peak value of cross-correlation (*N*=18) between the time series of the anterior-posterior motion of the pendulum and each body part by mobility (immobile vs. mobile) in gray and black dots and by frequency in the horizontal axis. Shown on the upper right of each panel is the result of the two-way analysis of variance: M is the main effect of the mobility factor, F is the main effect of the frequency factor, and M × F is the interaction of the mobility and frequency factors (p-value set at 0.05).

A two-way analysis of variance (ANOVA) on the first peak values of the cross-correlation showed that, in the in-phase condition, the main effect of the mobility factor was observed in all 18 combinations, and the correlation values in all combinations, except for R-pendulum and L-pendulum, were higher in the immobile condition than in the mobile condition (see Fig 4 for the results of motion similarity between the right pendulum and body parts, and see Table 1 for all of the results, including motion similarity between L-pendulum and body parts). Thus, the motion similarity between the pendulum and body parts was higher in the immobile condition than in the mobile condition, while that between pendulums was higher in the mobile condition than in the immobile condition.

**Table 1:** Results of two-way ANOVA for first peak value of cross-correlation (in-phase condition).

	R-pendulum	R-wrist	R-elbow	R-shoulder	C7
*RP*	No test conducted	*F*(1, 204) = 21.26, p<.001	*F*(1, 204) = 9.321, p = 0.003	*F*(1, 204) = 30.586, p<.001	*F*(1, 204) = 11.537, p<.001
		*F*(5, 204) = 0.535, p = 0.75	*F*(5, 204) = 0.469, p = 0.799	*F*(5, 204) = 2.903, p = 0.015	*F*(5, 204) = 2.945, p = 0.014
		*F*(5, 204) = 0.342, p = 0.887	*F*(5, 204) = 1.13, p = 0.346	*F*(5, 204) = 0.626, p = 0.68	*F*(5, 204) = 0.621, p = 0.684
*LP*	*F*(1, 204) = 7.045, p = 0.009	*F*(1, 204) = 22.76, p<.001	*F*(1, 204) = 7.864, p = 0.006	*F*(1, 204) = 30.557, p<.001	*F*(1, 204) = 11.076, p = 0.001
	*F*(5, 204) = 11.038, p<.001	*F*(5, 204) = 0.777, p = 0.568	*F*(5, 204) = 0.785, p = 0.561	*F*(5, 204) = 3.23, p = 0.008	*F*(5, 204) = 3.612, p = 0.004
	*F*(5, 204) = 0.369, p = 0.87	*F*(5, 204) = 0.404, p = 0.845	*F*(5, 204) = 1.064, p = 0.381	*F*(5, 204) = 0.784, p = 0.562	*F*(5, 204) = 0.839, p = 0.523
	L-pendulum	L-wrist	L-elbow	L-shoulder	Head
*RP*	*F*(1, 204) = 7.045, p = 0.009	*F*(1, 204) = 20.68, p<.001	*F*(1, 204) = 8.709, p = 0.004	*F*(1, 204) = 24.794, p<.001	*F*(1, 204) = 5.9, p = 0.016
	*F*(5, 204) = 11.038, p<.001	*F*(5, 204) = 0.619, p = 0.685	*F*(5, 204) = 1.683, p = 0.14	*F*(5, 204) = 2.749, p = 0.02	*F*(5, 204) = 1.153, p = 0.334
	*F*(5, 204) = 0.369, p = 0.87	*F*(5, 204) = 1.067, p = 0.38	*F*(5, 204) = 1.644, p = 0.15	*F*(5, 204) = 0.294, p = 0.916	*F*(5, 204) = 1.761, p = 0.123
*LP*	No test conducted	*F*(1, 204) = 16.928, p<.001	*F*(1, 204) = 7.171, p = 0.008	*F*(1, 204) = 20.502, p<.001	*F*(1, 204) = 5.695, p = 0.018
		*F*(5, 204) = 0.519, p = 0.762	*F*(5, 204) = 1.338, p = 0.249	*F*(5, 204) = 3.214, p = 0.008	*F*(5, 204) = 1.04, p = 0.395
		*F*(5, 204) = 0.756, p = 0.583	*F*(5, 204) = 1.39, p = 0.229	*F*(5, 204) = 0.456, p = 0.808	*F*(5, 204) = 1.73, p = 0.129

Note: First line: main effect of mobility; Second line: main effect of task frequency; Third line: interaction of two factors.

#### Anti-phase

**Fig 5:**
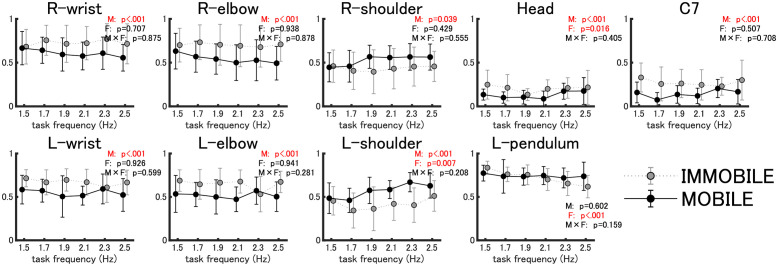
Motion similarity between the pendulum and body parts (anti-phase) (*N*=18). Fig 5 represent the same information as Fig 4, except that the data are for anti-phase.

A two-way ANOVA on the first peak values of the cross-correlation for the anti-phase condition showed the main effects of the mobility factor in 16 pairs, except for the pendulum. Post-hoc tests revealed that correlations of the R-pendulum with the right and left shoulders were higher in the mobile condition than in the immobile condition, and correlations of the R-pendulum with the rest of the body parts were higher in the immobile condition than in the mobile condition (see Fig 5 for results of the motion similarity between R-pendulum and body parts, and see Table 2 for all of the results, including motion similarity between the left pendulum and body parts). Thus, when the body is unconstrained, the similarity of pendulums to the shoulders (body parts two joints away from the wrists that are directly engaged in the pendulum swinging task) increases.

**Table 2:** Results of two-way ANOVA for first peak value of cross-correlation (anti-phase condition).

	R-pendulum	R-wrist	R-elbow	R-shoulder	C7
*RP*	No test conducted	*F*(1, 204) = 30.8, p<.001	*F*(1, 204) = 16.733, p<.001	*F*(1, 204) = 4.312, p = 0.039	*F*(1, 204) = 20.088, p<.001
		*F*(5, 204) = 0.591, p = 0.707	*F*(5, 204) = 0.252, p = 0.938	*F*(5, 204) = 0.983, p = 0.429	*F*(5, 204) = 0.863, p = 0.507
		*F*(5, 204) = 0.36, p = 0.875	*F*(5, 204) = 0.356, p = 0.878	*F*(5, 204) = 0.794, p = 0.555	*F*(5, 204) = 0.589, p = 0.708
*LP*	*F*(1, 204) = 0.273, p = 0.602	*F*(1, 204) = 33.216, p<.001	*F*(1, 204) = 18.807, p<.001	*F*(1, 204) = 41.797, p<.001	*F*(1, 204) = 16.66, p<.001
	*F*(5, 204) = 5.163, p<.001	*F*(5, 204) = 0.677, p = 0.641	*F*(5, 204) = 0.797, p = 0.553	*F*(5, 204) = 4.05, p = 0.002	*F*(5, 204) = 1.164, p = 0.328
	*F*(5, 204) = 1.611, p = 0.159	*F*(5, 204) = 0.618, p = 0.686	*F*(5, 204) = 0.15, p = 0.98	*F*(5, 204) = 2.706, p = 0.022	*F*(5, 204) = 0.91, p = 0.475
	L-pendulum	L-wrist	L-elbow	L-shoulder	Head
*RP*	*F*(1, 204) = 0.273, p = 0.602	*F*(1, 204) = 25.287, p<.001	*F*(1, 204) = 19.66, p<.001	*F*(1, 204) = 40.544, p<.001	*F*(1, 204) = 13.954, p<.001
	*F*(5, 204) = 5.163, p<.001	*F*(5, 204) = 0.276, p = 0.926	*F*(5, 204) = 0.247, p = 0.941	*F*(5, 204) = 3.304, p = 0.007	*F*(5, 204) = 2.874, p = 0.016
	*F*(5, 204) = 1.611, p = 0.159	*F*(5, 204) = 0.733, p = 0.599	*F*(5, 204) = 1.263, p = 0.281	*F*(5, 204) = 1.451, p = 0.208	*F*(5, 204) = 1.024, p = 0.405
*LP*	No test conducted	*F*(1, 204) = 22.417, p<.001	*F*(1, 204) = 23.812, p<.001	*F*(1, 204) = 4.803, p = 0.03	*F*(1, 204) = 8.185, p = 0.005
		*F*(5, 204) = 0.579, p = 0.716	*F*(5, 204) = 0.462, p = 0.804	*F*(5, 204) = 0.791, p = 0.557	*F*(5, 204) = 2.017, p = 0.078
		*F*(5, 204) = 1.073, p = 0.376	*F*(5, 204) = 1.93, p = 0.091	*F*(5, 204) = 0.49, p = 0.783	*F*(5, 204) = 0.79, p = 0.558

Note: First line: main effect of mobility; Second line: main effect of task frequency; Third line: interaction of two factors.

### Phase difference between the pendulum and body parts

#### In-phase

**Fig 6:**
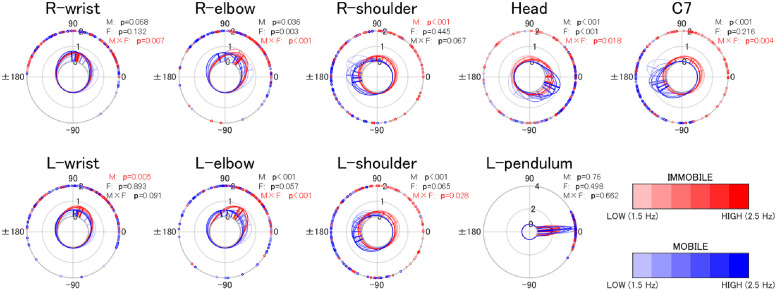
Phase difference between the pendulum and body parts (in-phase) (*N*=18). This shows the relative phase between the anterior-posterior motion of the pendulum and each body part (*N*=18) by mobility (immobile vs. mobile) in color temperature (warm vs. cold) and frequency as color gradient. All data of the 18 participants are shown in the outer circle. Two things are shown on the inner circle: the mean vector (straight line) of the data of all participants, of which the length corresponds to the angle concentration *κ*, and the probability density function (von Mises distribution) calculated based on the mean vector and concentration *κ*. Results of the two-way Harrison-Kanji test are shown on the upper right of each panel.

Results of the two-way Harrison-Kanji test for the mean of the relative phase showed that, in the in-phase condition, there was an interaction between mobility and frequency for the combinations of R-wrist, R-elbow, L-elbow, L-shoulder, head, and C7 with L- and R-pendulums (12 pairs; Fig 6; see Table 3). Results of the simple main effect tests showed that the frequency’s main effect was observed only in the mobile condition for body parts in the immediate vicinity of the pendulum (R-wrist, R-elbow, and L-elbow). That is, changes in the phase differences between the pendulums and these body parts (R-wrist, R-elbow, and L-elbow) depend on the change in frequency only when the entire body is unconstrained.

**Table 3:** Results of the two-way Harrison-Kanji test for phase differences (in-phase condition).

	R-pendulum	R-wrist	R-elbow	R-shoulder	C7
*RP*	No test conducted	*χ* 2(2) = 5.381, p = 0.068	*χ* 2(2) = 6.661, p = 0.036	*χ* 2(2) = 104.761, p<.001	*χ* 2(2) = 134.189, p<.001
		*χ* 2(10) = 15.011, p = 0.132	*χ* 2(10) = 26.929, p = 0.003	*χ* 2(10) = 9.954, p = 0.445	*χ* 2(10) = 13.137, p = 0.216
		*χ* 2(5) = 15.887, p = 0.007	*χ* 2(5) = 31.017, p<.001	*χ* 2(5) = 10.321, p = 0.067	*χ* 2(5) = 17.273, p = 0.004
*LP*	*F*(1, 204) = 0.093, p = 0.76	*χ* 2(2) = 5.687, p = 0.058	*χ* 2(2) = 5.589, p = 0.061	*χ* 2(2) = 94.196, p<.001	*χ* 2(2) = 129.655, p<.001
	*F*(5, 204) = 0.877, p = 0.498	*χ* 2(10) = 15.348, p = 0.12	*χ* 2(10) = 26.191, p = 0.003	*χ* 2(10) = 10.336, p = 0.412	*χ* 2(10) = 14.228, p = 0.163
	*F*(5, 204) = 0.649, p = 0.662	*χ* 2(5) = 16.413, p = 0.006	*χ* 2(5) = 25.543, p<.001	*χ* 2(5) = 7.606, p = 0.179	*χ* 2(5) = 14.985, p = 0.01
	L-pendulum	L-wrist	L-elbow	L-shoulder	Head
*RP*	*F*(1, 204) = 0.093, p = 0.76	*χ* 2(2) = 10.659, p = 0.005	*χ* 2(2) = 20.749, p<.001	*χ* 2(2) = 80.855, p<.001	*χ* 2(2) = 43.676, p<.001
	*F*(5, 204) = 0.877, p = 0.498	*χ* 2(10) = 4.973, p = 0.893	*χ* 2(10) = 17.909, p = 0.057	*χ* 2(10) = 17.428, p = 0.065	*χ* 2(10) = 65.888, p<.001
	*F*(5, 204) = 0.649, p = 0.662	*χ* 2(5) = 9.499, p = 0.091	*χ* 2(5) = 21.244, p<.001	*χ* 2(5) = 12.506, p = 0.028	*χ* 2(5) = 13.673, p = 0.018
*LP*	No test conducted	*χ* 2(2) = 7.333, p = 0.026	*χ* 2(2) = 20.055, p<.001	*χ* 2(2) = 89.425, p<.001	*χ* 2(2) = 38.813, p<.001
		*χ* 2(10) = 6.186, p = 0.799	*χ* 2(10) = 22.194, p = 0.014	*χ* 2(10) = 15.353, p = 0.12	*χ* 2(10) = 59.661, p<.001
		*χ* 2(5) = 10.228, p = 0.069	*χ* 2(5) = 23.958, p<.001	*χ* 2(5) = 11.875, p = 0.037	*χ* 2(5) = 12.346, p = 0.03

Note: First line: main effect of mobility; Second line: main effect of task frequency; Third line: interaction of two factors.

As for body parts remote from the wrists directly engaged in the task (head, C7, and shoulder), the frequency’s main effect was significant in both the immobile and mobile conditions, and the main effect of mobility factors was observed in each frequency. In other words, for the mobile and immobile conditions, the phase difference between the pendulums and the remote body parts varied with frequency, and the pattern of variation was qualitatively different in the mobility factor.

#### Anti-phase

**Fig 7:**
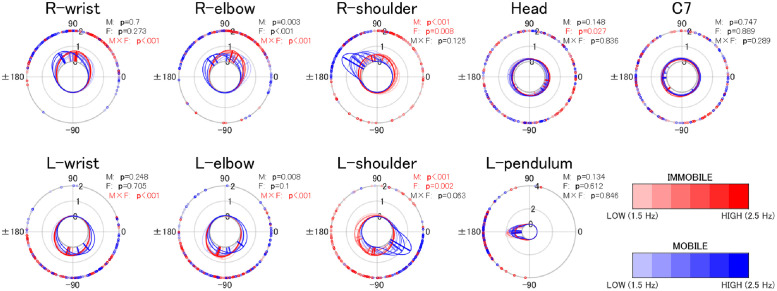
Phase difference between the pendulum and body parts (anti-phase) (*N*=18). Fig 7 represent the same information as Fig 6, except that the data are for anti-phase.

In the anti-phase condition, interactions were observed between mobility and frequency for body parts in the immediate vicinity of the pendulum (R-wrist, L-wrist, R-elbow, and L-elbow, i.e., 8 pairs; Fig 7; Table 4). A simple main effect test showed that the frequency’s main effect was observed in only the mobile condition for all combinations. In other words, as in the in-phase condition, phase differences between the pendulums and these body parts (R-wrist, L-wrist, R-elbow, and L-elbow) depend on frequency only when the entire body is unconstrained. As for body parts remote from the wrists, namely, R-shoulder and L-shoulder, main effects of both the mobility and frequency factors were observed. In these parts, frequency-dependent phase difference patterns were observed in both the mobile and immobile conditions, and these patterns in both conditions were qualitatively different.

**Table 4:** Results of the two-way Harrison-Kanji test for phase differences (anti-phase condition).

	R-pendulum	R-wrist	R-elbow	R-shoulder	C7
*RP*	No test conducted	*χ* 2(2) = 0.714, p = 0.7	*χ* 2(2) = 11.521, p = 0.003	*χ* 2(2) = 94.218, p<.001	*χ* 2(2) = 0.584, p = 0.747
		*χ* 2(10) = 12.182, p = 0.273	*χ* 2(10) = 31.471, p<.001	*χ* 2(10) = 23.983, p = 0.008	*χ* 2(10) = 5.031, p = 0.889
		*χ* 2(5) = 28.198, p<.001	*χ* 2(5) = 37.299, p<.001	*χ* 2(5) = 8.62, p = 0.125	*χ* 2(5) = 6.179, p = 0.289
*LP*	*F*(1, 204) = 2.265, p = 0.134	*χ* 2(2) = 2.145, p = 0.342	*χ* 2(2) = 16.67, p<.001	*χ* 2(2) = 83.713, p<.001	*χ* 2(2) = 4.006, p = 0.135
	*F*(5, 204) = 0.716, p = 0.612	*χ* 2(10) = 10.938, p = 0.362	*χ* 2(10) = 31.159, p<.001	*χ* 2(10) = 26.341, p = 0.003	*χ* 2(10) = 3.191, p = 0.977
	*F*(5, 204) = 0.403, p = 0.846	*χ* 2(5) = 21.987, p<.001	*χ* 2(5) = 28.106, p<.001	*χ* 2(5) = 9.401, p = 0.094	*χ* 2(5) = 9.587, p = 0.088
	L-pendulum	L-wrist	L-elbow	L-shoulder	Head
*RP*	*F*(1, 204) = 2.265, p = 0.134	*χ* 2(2) = 2.79, p = 0.248	*χ* 2(2) = 9.7, p = 0.008	*χ* 2(2) = 100.782, p<.001	*χ* 2(2) = 3.823, p = 0.148
	*F*(5, 204) = 0.716, p = 0.612	*χ* 2(10) = 7.218, p = 0.705	*χ* 2(10) = 16.002, p = 0.1	*χ* 2(10) = 27.152, p = 0.002	*χ* 2(10) = 20.235, p = 0.027
	*F*(5, 204) = 0.403, p = 0.846	*χ* 2(5) = 22.105, p<.001	*χ* 2(5) = 22.709, p<.001	*χ* 2(5) = 10.474, p = 0.063	*χ* 2(5) = 2.095, p = 0.836
*LP*	No test conducted	*χ* 2(2) = 5.264, p = 0.072	*F*(1, 204) = 12.61, p<.001	*χ* 2(2) = 104.984, p<.001	*χ* 2(2) = 9.713, p = 0.008
		*χ* 2(10) = 12.182, p = 0.273	*F*(5, 204) = 2.991, p = 0.013	*χ* 2(10) = 35.329, p<.001	*χ* 2(10) = 10.757, p = 0.377
		*χ* 2(5) = 26.768, p<.001	*F*(5, 204) = 3.904, p = 0.002	*χ* 2(5) = 15.649, p = 0.008	*χ* 2(5) = 4.277, p = 0.51

Note: First line: main effect of mobility; Second line: main effect of task frequency; Third line: interaction of two factors.

More specifically, phase difference between the pendulums and shoulders in the mobile condition underwent counterclockwise transitions as the frequency increased (note that the deeper the color, the higher the frequency). This corresponds to the counterclockwise transitions in the phase differences between the pendulum and the body parts in the vicinity of the pendulum (i.e., wrists and elbows) as the frequency increases. This can be interpreted as an effect of the mechanical connections among the body parts. Furthermore, in the anti-phase condition, unlike in the in-phase condition, no main effect or interaction was observed for the head or C7. In other words, we cannot say that the phase difference between the head and C7 changed depending on the frequency or mobility factors.

### Degree of task achievement

#### In-phase

**Fig 8:**
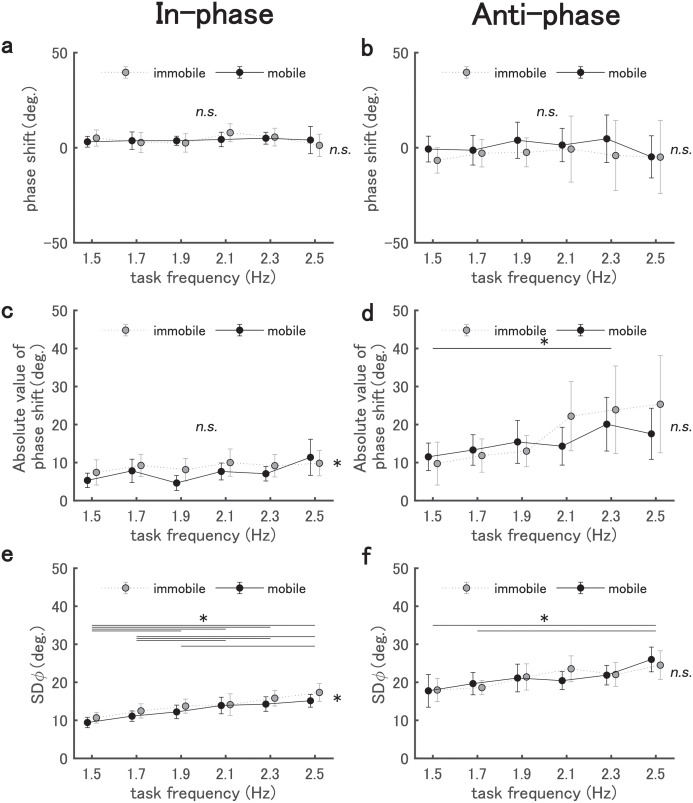
Results of the phase difference between the left-right pendulum. The phase shift (panels (a) and (b)), absolute value of the phase shift (panels (c) and (d)), and SD*ϕ* (Fig 8e and 8f) are shown for each phase mode (in-phase and anti-phase). Line plots with markers indicate the mean values for 18 participants, and error bars indicate 95% confidence intervals. Asterisks represent pairs that were significant in the Watson-Williams test (performed as a post-hoc test when the two-factor Harrison-Kanji test was significant).

Results of the two-way Harrison-Kanji test conducted on the phase shift and absolute phase shift for mobility and frequency in the in-phase mode condition are shown in Fig 8a, 8c and 8e): While the phase shift (Fig 8a) had no significant main effect and no interaction, absolute value of the phase shift (Fig 8c) had significant main effects for both mobility and frequency, Mobility: *F*(1, 204) = 4.527, *p*=0.035; Frequency: *F*(5, 204) = 2.527, *p*=0.03. Post-hoc tests revealed that the absolute value of the phase shift was significantly smaller in the mobile condition than in the immobile condition, and no significant difference was confirmed for frequency in any pair (*adjusted p*>.05). These results show that the participants were better in an attempt to maintain the in-phase mode in the mobile condition than in the immobile condition.

For the SD*ϕ* (Fig 8e), while the interaction between mobility and frequency was not significant, the main effect was significant for both factors, Mobility: *F*(1, 204) = 9.586, *p*=0.002; Frequency: *F*(5, 204) = 13.227, *p*<.001. Post-hoc tests showed that SD*ϕ* was significantly smaller in the mobile condition than in the immobile condition. It also showed that SD*ϕ* was significantly different in the following comparisons: 1.5, 1.7 Hz < 1.9, 2.1, 2.3, 2.5 Hz, and 1.9 Hz < 2.5 Hz (adjusted *p*<.05). These results imply that while the task performance in keeping the in-phase mode became increasingly unstable as the frequency increased, it was achieved more stably in the mobile condition than in the immobile condition.

#### Anti-phase

Results of the two-way Harrison-Kanji test for mobility and frequency for the anti-phase mode are shown in Fig 8b, 8d and 8f). While there was no significant main effect or interaction for the phase shift (Fig 8b), the absolute value of the phase shift (Fig 8d) exhibited a significant main effect for frequency, *F*(5, 204) = 3.912, *p*=0.002. The post-hoc test showed that the absolute value of phase shift was significantly smaller in the 1.5 Hz condition than in the 2.3 Hz condition (*adjusted p*<.05). Therefore, the degree of deviation from the targeted phase mode, that is, anti-phase, increased with frequency. For the SD*ϕ* (Fig 8f), there was a significant main effect for the frequency factor, *F*(5, 204) = 5.209, *p*<.001. The post-hoc test showed that SD*ϕ* was significantly larger in the 2.5 Hz condition than in the 1.5 Hz and 1.7 Hz conditions (*adjusted p*<.05). Collectively, as in the in-phase condition, the phase difference became more unstable as the frequency increased, but it did not change regardless of mobility.

## Discussion

Here, we put the results into perspective and discuss whether and how global coordinative structures emerged under the conditions set in this study. Let us start by confirming the constraints in different conditions set for the present experiment.

Now, in the immobile condition, in comparison with the mobile condition, the following were found: regardless of the phase mode, all of the body parts, except for shoulders in the anti-phase condition, exhibited a higher degree of motion similarity with the right pendulum (Figs 2 and 3; Tables 1 and 2), and the frequency-dependent phase difference changes were shown to be smaller for the body parts in the vicinity of the pendulum (wrist and elbow; Figs 4 and 5).

In the mobile condition, there are only three surfaces of contact with the environment: the buttocks and a pair of soles; thus, the whole body is less stable than in the immobile condition with five surfaces of support: the buttocks, a pair of soles and the forearms. Accordingly, a more compensatory motion is required to perform the pendulum task in the mobile condition than in the immobile condition. However, because there were more degrees of freedom in the mobile condition, it is possible that the participants were able to use them to regulate the whole body to achieve the pendulum task while maintaining the posture. The way in which the participants used the degrees of freedom seems to depend on the phase mode, and different patterns seem to have emerged.

Given the above consideration, the higher degree of similarity in motion between the body parts and the pendulums in the immobile condition can be interpreted as an emerging coordinative structure in which the impact of the pendulums is dispersed and absorbed by the five surfaces of support. Phase differences did not change significantly regardless of frequency, because the whole body was more stable with more support surfaces in this condition than in the immobile condition. That is, the demand to compensate for the movement of the pendulum weight to maintain the sitting position may have been much less in the immobile condition than in the mobile condition.

Note that these findings of higher similarity in motion between the pendulums and most of the body parts were obtained under the condition wherein the forearms were tightly fastened to the tabletop. That is, the table and the forearms as a whole might have moved more or less together by swinging the pendulums.

Meanwhile, in the mobile condition, we observed characteristic coordination patterns for both the frequency and phase modes.

**Fig 9:**
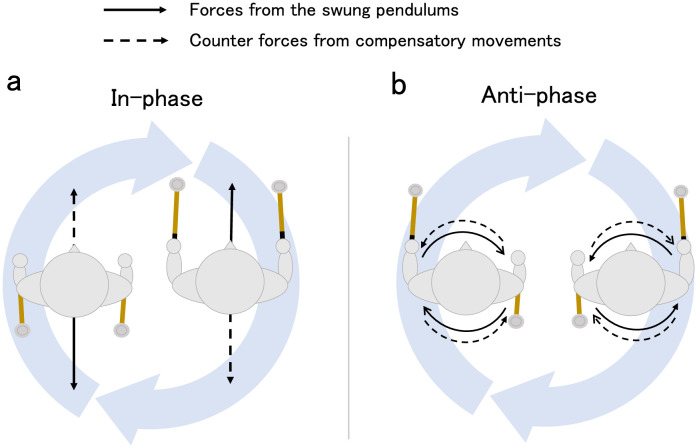
Schematic diagram of possible forces at work and compensatory movements during the present experiment by phase mode. Solid lines indicate the direction of forces caused by swinging the pendulums, which may affect the body, and dashed lines indicate the direction of counter forces caused by possible compensatory movements. This study’s results appear to correspond to the compensatory movements shown in this diagram.

As for the in-phase condition in the mobile condition, the remote body parts (shoulder and C7) and the pendulums were in anti-phase (phase differences were in the range of 170–180 degrees; Fig 4), suggesting the emergence of a coordinative structure that counterbalanced the swinging mass of the pendulum (see Fig 9a). In the in-phase condition, the two pendulums in pairs moved alternately toward and away from the front of the participant’s body, and it was necessary for the participant to compensate for the impact of the moving weight to maintain the posture (see Fig 9a). To achieve this, the participants might have had to move their remote body parts (shoulder and C7) alternatingly to the moving pendulums on the anterior-posterior axis. The results of the anti-phase between the pendulum and the distal body part in the conditions under discussion can be interpreted as a result of meeting these requirements.

Interestingly, despite the consistent anti-phase relation between the pendulums and the shoulders/C7s, phase differences between the pendulum and the head varied as a function of frequency from 130 degrees (1.5 Hz) to 20 degrees (2.5 Hz; Fig 6). This suggests that the trunk and head are segmented in the high-frequency condition (2.5 Hz) compared with the low-frequency condition (Fig 6). A similar pattern was observed in the immobile condition, although the amplitude of the change was smaller than that in the mobile condition. These phenomena may be considered adaptive behavior to cope with the effect that, as frequency increases, muscle stiffness increases, resulting in a decrease in the dampening capacity in the pendulum-swinging task (Hamaoui et al. [18, 19] found that the passive dampening capacity of the postural chain decreases as muscle stiffness increases).

As for the anti-phase mobile condition, the following were observed: The shoulders, but not others, showed higher similarity in motion to the pendulum compared with the immobile condition (Fig 3). Frequency-dependent changes in phase difference at the body parts close to the pendulum (wrist and elbow) were observed only in the mobile condition (Fig 5). Among body parts remote to the pendulum (shoulder, C7, and head), phase differences between the shoulder and pendulum of the same side (e.g., R-pendulum and R-shoulder) were in anti-phase, and those of the opposite side (e.g., R-pendulum and L-shoulder) were in the in-phase as frequency increased in the mobile condition.

In the anti-phase condition, the movement of two pendulums in opposite directions at a distance of the shoulder width in effect rotates the torso, as shown in Fig 9b. To keep the posture, the participant must compensate for the rotation force. That is, given that the phase differences between the shoulders and the pendulum of the same side and between those of the opposite side were clearly different (Fig 7), the Head and C7 were not similar to the pendulum (Fig 3), and phase differences were not consistent (Fig 5), the torso together with the shoulders rotated with the Head and C7 as the axis of rotation and a coordinative structure to cope with the rotational force to the body from moving the pendulums in the opposite directions in the anti-phase condition (See Fig 9b).

Taken together, when the body was unconstrained to move, a global coordinative structure emerged according to the demands of the in-phase and anti-phase conditions, respectively. Let us now discuss the global coordinative structure in relation to the task achievement.

As described by the HKB model [3], the position of a basin (attractor) in the attractor landscape can be estimated from phase variables (mean*ϕ*, phase shift, etc.) and the depth using SD*ϕ*, etc [1, 3, 20]. For example, if SD*ϕ* is larger, the basin of attraction can be estimated to be shallower.

Given that both SD*ϕ* and the phase shift were smaller in the in-phase mobile condition than the immobile condition, we can infer in dynamical terms that there was an attractor common to the participants, and that it was deeper as well as closer to the prescribed relative phase in the in-phase mobile condition than in the in-phase immobile condition.

As for the anti-phase condition, while SD*ϕ* was slightly larger than that of the in-phase condition as Fig 8e and 8f show, there was no statistical difference in mobility in either phase shift or SD*ϕ*. This suggests that the attractor depth was equivalent in both mobility conditions. Additionally, given that the inter-participant variability was larger in the immobile condition than in the mobile condition, the position of an attractor between participants may well have been more diverse in the immobile condition than in the mobile condition, but at the equivalent depth. Thus, in light of the HKB dynamics, in the anti-phase immobile condition it is possible that there was no common strong attractor to all participants, with instead a different attractor for different individuals, in contrast to the anti-phase mobile condition. In such a case, the underlying dynamics may possibly be different for the two conditions.

In the present study, we obtained findings suggestive of extending the conventional model of inter-limb coordination (i.e., the HKB model) to more practical motor tasks. That is, in the mobile condition, while the focal phase difference (in-phase or anti-phase) was maintained (suggesting the HKB model’s validity), the phase difference between the torso and the pendulum exhibited an anti-phase pattern.

Although the anti-phase has been characterized as a less stable state in the conventional two-oscillator model, it may be considered an important state that supports focal stability in the background when viewed from the perspective of the whole-body coordinative structure, including posture.

The idea of “beyond HKB” may not be very new [10, 21, 22], but by representing the equilibrium state of the whole body as an attractor depending on various conditions, and by clarifying the correspondence with focal performance, we may construct a more practical model that goes beyond the conventional two oscillator scale.

Finally, we discuss the implications of the research on musical performance. In this study, we confirmed that the global coordinative structure varies with the task-dependent states of the local body parts (e.g., in-phase or anti-phase).

As alluded to earlier, in musical instrument performance, motion of the local body parts in direct contact with the instrument can be considered as essentially inseparable from the postural control of the whole body. Then, how are the local body parts directly involved in producing sound coordinated with the maintenance of posture? How is coordination regulated by the characteristics of the instrument and the composition of music? Do these depend on the skill level?

Attempts have been made to answer some of these questions. For example, a study on drum-set playing [23] showed that phase differences between the right wrist, which is close to the hand holding a stick, and other remote body parts were calculated and compared between participants and tempi for professional drummers. The phase difference between the wrist and other body parts changed adaptively with the tempo, and different drummers exhibited unique patterns when the tempi were set to slow or normal, although the accuracy of the tapping did not drastically differ across the conditions and among drummers.

Similarly, in research on violin [24] and cello [25], the relationship between the elbow, which is involved in the bowing motion, and other body parts has been quantified and compared between skilled and novice players. These studies suggest that movement of the shoulder is suppressed in relation to the elbow in skilled players, whereas in novices, the shoulder is not well controlled. In other words, the range of motion of the elbow is much wider than that of the shoulder.

Some coordinative structures specific to instrumental playing can be only revealed by looking at local body parts. To truly understand the mechanism of instrumental performance, it is essential to explore the instrument/expert-specific structures that emerge in the global coordinative structure in relation to the local coordinative structure.
